# Review of Fabrication Methods, Physical Properties, and Applications of Nanostructured Copper Oxides Formed via Electrochemical Oxidation

**DOI:** 10.3390/nano8060379

**Published:** 2018-05-29

**Authors:** Wojciech J. Stepniowski, Wojciech Z. Misiolek

**Affiliations:** 1 Materials Science and Engineering Department & Loewy Institute, Lehigh University, 5 East Packer Ave., Bethlehem, PA 18015, USA; wzm2@lehigh.edu; 2 Department of Advanced Materials and Technologies, Faculty of Advanced Technology and Chemistry, Military University of Technology, Urbanowicza 2 Str., 00-908 Warszawa, Poland

**Keywords:** anodization, copper oxides, nanostructures, passivation, nanowires, nanoneedles, band gap

## Abstract

Typically, anodic oxidation of metals results in the formation of hexagonally arranged nanoporous or nanotubular oxide, with a specific oxidation state of the transition metal. Recently, the majority of transition metals have been anodized; however, the formation of copper oxides by electrochemical oxidation is yet unexplored and offers numerous, unique properties and applications. Nanowires formed by copper electrochemical oxidation are crystalline and composed of cuprous (CuO) or cupric oxide (Cu_2_O), bringing varied physical and chemical properties to the nanostructured morphology and different band gaps: 1.44 and 2.22 eV, respectively. According to its Pourbaix (potential-pH) diagram, the passivity of copper occurs at ambient and alkaline pH. In order to grow oxide nanostructures on copper, alkaline electrolytes like NaOH and KOH are used. To date, no systemic study has yet been reported on the influence of the operating conditions, such as the type of electrolyte, its temperature, and applied potential, on the morphology of the grown nanostructures. However, the numerous reports gathered in this paper will provide a certain view on the matter. After passivation, the formed nanostructures can be also post-treated. Post-treatments employ calcinations or chemical reactions, including the chemical reduction of the grown oxides. Nanostructures made of CuO or Cu_2_O have a broad range of potential applications. On one hand, with the use of surface morphology, the wetting contact angle is tuned. On the other hand, the chemical composition (pure Cu_2_O) and high surface area make such materials attractive for renewable energy harvesting, including water splitting. While compared to other fabrication techniques, self-organized anodization is a facile, easy to scale-up, time-efficient approach, providing high-aspect ratio one-dimensional (1D) nanostructures. Despite these advantages, there are still numerous challenges that have to be faced, including the strict control of the chemical composition and morphology of the grown nanostructures, their uniformity, and understanding the mechanism of their growth.

## 1. Introduction

Nanostructured anodic oxides have attracted the attention of researchers due to their ease of fabrication and tailored ordered morphology on the nanometric scale. What is more, the resulting chemical and physical properties lead to numerous potential applications [1]. The most frequently studied anodic oxides are hexagonally arranged anodic aluminum oxide (AAO) [1] and nanoporous or nanotubular anodic titanium oxide (ATO) [2]. Intensive research on those two nanostructured materials has incited significant progress in: electrochemical and optical sensing [3,4], nanofabrication [5], photonic crystals [6], information optical coding [7], filtration and kidney dialysis [8], drug releasing platforms [9], biomaterials performance [10], renewable energy harvesting [11,12], the removal of greenhouse gases [13], magnetic materials [14], surface-enhanced Raman spectroscopy [15], plasmonic materials [16], structural color generation [17], tunable contact angle surfaces [18,19], and tunable band gap materials [20].

Currently, the majority of transition metals have been tested as substrates for anodizing. Researchers have obtained nanostructured anodic oxides as the result of the electrochemical oxidation of: W [21,22], Sn [23], Zr [24,25], Zn [26,27], Nb [28,29], Fe [30], and FeAl [20,31]. The majority of the nanostructures obtained by transition metals anodization are composed of oxide, where the metallic element is at one fixed oxidation state. Furthermore, almost all of the oxides are nanoporous or nanotubular. So far, the only reported exception is anodically grown ZnO: in this case, the grown oxide is made of nanowires [26,27].

Another promising metal for oxide nanostructures fabrication via self-organized anodization is copper. Copper forms two oxides, namely cuprous oxide Cu_2_O and cupric oxide, CuO, as well as their mixtures in various phases such as copper-rich Cu_4_O_3_ [32]. A demand for the simple synthesis of copper oxides’ nanostructures is a result of the electronic properties of Cu_2_O, CuO, and Cu_4_O_3_. CuO is reported to be a p-type semiconductor with a band gap in the range from 1.2 to 2.16 eV, depending on the nature of the band gap (direct, or indirect), doping, morphology, and crystal size [32]. For CuO nanostructures, the smaller the size of the structure, the greater the band gap, observed as a blue shift in the spectrum. It is worth noting that Bohr’s radius for CuO is ca. 6.6 nm, thus below this size a strong quantum confinement (QC) is observed [32] (nevertheless, QC is also observed above 6.6 nm, although the phenomenon is much weaker). Cu_2_O is also a p-type semiconductor with a band gap over 2.1 eV [32]. However, also in this case, due to the QC, the band gap can be engineered and controlled during the manufacturing process. For example, a decrease of Cu_2_O film thickness from 5.4 to 0.75 nm increases the band gap from 2.6 to 3.8 eV [33]. Additionally, according to Musselman et al., Cu_2_O, due to the band gap value around 2.0 eV and theoretical maximum power conversion efficiency of approximately 20% (PCE), is suitable for applications in heterojunction solar cells [34]. 

There are numerous methods of Cu_2_O and CuO nanostructures fabrication, employing diverse chemical methods including micelle-assisted precipitation, sol-gel methods, and high-temperature annealing in an oxidative atmosphere. The formation of a wide range of nanostructures such as nanospheres, nanoflowers, leaf-shaped nanocrystals, nanorings, nanoribbons, multi-pod nanocrystals, etc. has been already reported and broad reviews reporting the state-of-the-art have been published [35,36]. Furthermore, this variety of nanostructures with high surface-to-bulk atom ratios has triggered research on numerous applications of Cu_2_O and CuO nanostructures, such as high-surface area electrode materials in batteries, photocatalysts in water purification systems, carbon dioxide reduction and water splitting, high-contact angle functional surfaces, gas sensors, infrared radiation sensors, etc. [35,36].

However, the anodization of copper, leading to the formation of nanostructures, has not been as intensively explored as other methods and was not even included in the numerous review reports focusing on the formation of cupric and cuprous oxide nanostructures. However, copper anodization may bring unexpected benefits in terms of morphology control, the formation of high-aspect ratio nanostructures, and their doping. Morphological features of nanostructured anodic oxides are tailored by operating conditions. For example, the pore diameter and intepore distance of anodic alumina and titania are linear functions of the applied voltage [1,2,20]. Additionally, electrochemical in situ doping of anodic oxides, due to the application of various additives in the electrolyte, allows one to dope the growing nanostructures [37]. Therefore, self-organized anodization seems to be a promising method in copper oxides formation, providing high-surface area nanostructures with a band gap tunable by operating conditions (size of the nanostructures) and chemical composition (in situ doping). Per analogiam to the anodization of Al and Ti [1,2,3,4,5,6,7,8,9,10,11,12,13,14,15,16,17,18,19,20,21,22,23,24,25,26,27,28,29,30,31,32,33,34,35,36,37], it can be expected that the morphology of the nanostructures formed by copper anodization can be tailored after the optimization of the operating conditions. Moreover, recent advances in Al and Ti anodization, as well as recent high-tech applications, may provide some inspiration for electrochemical copper oxidation.

## 2. Passivation of Copper

**Figure 1:**
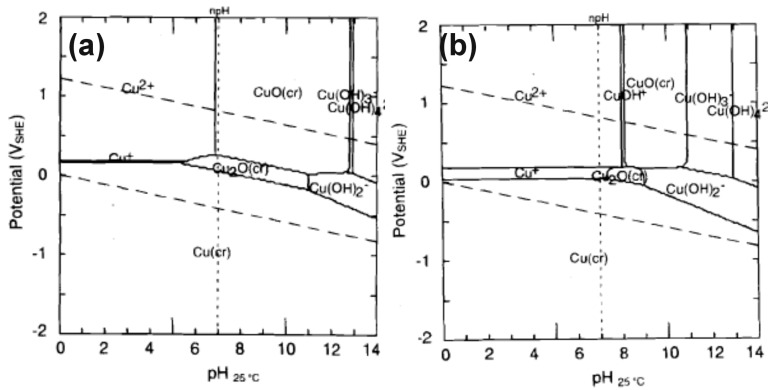
Pourbaix diagram for copper at 25 °C for total copper species concentration in a solution equal to 10^−6^ mol/L (**a**) and 10^−8^ mol/L (**b**). Reproduced with permission from [38]. Electrochemical Society, 1997.

Pourbaix diagrams (potential vs. pH diagram) provide key information for understanding the electrochemical oxidation of metals. According to the Pourbaix diagram for copper, it is apparent that the most suitable electrolytes for copper anodization are the alkaline ones (solutions of bases like NaOH, KOH, but also salts with alkaline hydrolysis like carbonates and bicarbonates of potassium and sodium, not researched yet as potential copper anodizing electrolytes) (Figure 1) [38]. It is also noticeable that electrochemical oxidation may lead to the formation of oxides like Cu_2_O (lower potentials) and CuO (greater potentials), as well as cupric hydroxide Cu(OH)_2_ and water-soluble coordination anions with hydroxyl ligands, namely Cu(OH)_3_^−^ and Cu(OH)_4_^2−^. For anodizing, the minimal solubility of Cu_2_O in water is at a pH range from 7.5 to 8.0 [38]. It is also worth noting that the as-obtained anodic oxides formed on copper are crystalline, while other anodic oxides, like titania or alumina, are amorphous. The formation of crystalline phases in anodic alumina or titania requires annealing after the anodization.

Due to the formation of two copper oxides and the formation of soluble coordination ions, the mechanism of copper anodization is much more complex than in the case of other oxides, like AAO. Gennero de Chiavlo et al. analyzed the oxidation of copper only to the Cu^+^ oxidation state and, already at this stage, the occurring phenomena are complex [39]. According to the chemical reactions, first copper oxidizes and forms metastable CuOH on the Cu surface (1):

(1) $$\mathrm{Cu}+{\mathrm{OH}}^{-}\to \mathrm{CuOH}+\;\bar{e}$$

Next, cuprous hydroxide, CuOH, may react in two ways, either forming solid Cu_2_O (2):

(2) $$2\mathrm{CuOH}\to {\mathrm{Cu}}_{2}O+H_{2}O$$

Or bonding the hydroxyl group and forming water-soluble Cu_2_O_2_H^−^ (3):

(3) $$2\mathrm{CuOH}+{\mathrm{OH}}^{-}\to {\mathrm{Cu}}_{2}O_{2}H^{-}+H_{2}O$$

In an alkaline environment, Cu_2_O_2_H^−^ may also transform easily into Cu_2_O_2_^2−^, when OH^−^ accepts a proton.

The cuprous oxide, under the influence of hydroxyl anions, may transform into the water-soluble Cu_2_O_2_^2−^ (4):

(4) $${\mathrm{Cu}}_{2}O+2{\mathrm{OH}}^{-}\to {\mathrm{Cu}}_{2}O_{2}^{2-}+H_{2}O$$

Furthermore, the formed Cu_2_O_2_^2−^, anions are metastable and disproportionate into solid, metallic Cu and water-soluble CuO_2_^2−^, which can be also considered as [Cu(OH)_4_]^2−^ (5):

(5) $${\mathrm{Cu}}_{2}O_{2}^{2-}\to {\mathrm{CuO}}_{2}^{2-}+\mathrm{Cu}$$

Thus, the re-deposited copper may undergo the entire cycle of reactions again, starting from Reaction (1). According to Ambrose et al., and their voltammetric study of Cu in KOH, a water-soluble Cu(I) species may also be formed directly from Cu, due to the formation of a coordination anion (6) [40]:

(6) $$\mathrm{Cu}+2{\mathrm{OH}}^{-}\to \mathrm{Cu}{\left(\mathrm{OH}\right)}_{2}^{-}+\;\bar{e}\;\mathrm{Ep}\;=\;-550\;\mathrm{mV}\;\mathrm{vs}.\;\mathrm{Hg}|\mathrm{HgO}$$

However, according to Reference [40], Cu_2_O may also be electrochemically formed by the following Reaction (7):

(7) $$2\mathrm{Cu}+2{\mathrm{OH}}^{-}\to {\mathrm{Cu}}_{2}O+H_{2}O+2\bar{e}\;\mathrm{Ep}\;=\;-400\;\mathrm{mV}\;\mathrm{vs}.\;\mathrm{Hg}|\mathrm{HgO}$$

The mechanism becomes more complex when the formation of Cu(II) from metallic Cu and Cu(I) is considered. Copper may oxidize directly to the soluble species like the coordination anion (8) [40]:

(8) $$\mathrm{Cu}+4{\mathrm{OH}}^{-}\to \mathrm{Cu}{\left(\mathrm{OH}\right)}_{4}^{2-}+2\bar{e}\;\mathrm{Ep}\;=-100\;\mathrm{mV}\;\mathrm{vs}.\;\mathrm{Hg}|\mathrm{HgO}$$

However, the already-grown cuprous oxide, Cu_2_O, may undergo further oxidation to the abovementioned soluble species (Equations (4) and (5)), or may form insoluble cupric hydroxide:

(9) $${\mathrm{Cu}}_{2}O+2{\mathrm{OH}}^{-}+H_{2}O\to 2\mathrm{Cu}{\left(\mathrm{OH}\right)}_{2}+2\bar{e}\;\mathrm{Ep}\;=-100\;\mathrm{mV}\;\mathrm{vs}.\;\mathrm{Hg}|\mathrm{HgO}$$

Nevertheless, this Cu(OH)_2_ deposit may form a soluble species like [Cu(OH)_4_]^2−^ in an alkaline environment [40]. According to the fundamental work by Ambrose et al. [40], at greater potentials Cu(II) may be directly formed by copper oxidation, according to the following reactions (10) and (11):

(10) $$\;\mathrm{Cu}+2{\mathrm{OH}}^{-}\to \mathrm{Cu}{\left(\mathrm{OH}\right)}_{2}+2\bar{e}\;\mathrm{Ep}\;=0\;\mathrm{mV}\;\mathrm{vs}.\;\mathrm{Hg}|\mathrm{HgO}$$

(11) $$\mathrm{Cu}+2{\mathrm{OH}}^{-}\to \mathrm{CuO}+H_{2}O+2\bar{e}\;\mathrm{Ep}\;=\;0\;\mathrm{mV}\;\mathrm{vs}.\;\mathrm{Hg}|\mathrm{HgO}\;$$

However, CuO and Cu(OH)_2_ may also transform into soluble species, with copper at a greater oxidation state, namely Cu(III), when sufficiently high potentials are applied, as in reactions (12) and (13) [40]:

(12) $$\mathrm{Cu}{\left(\mathrm{OH}\right)}_{2}+2{\mathrm{OH}}^{-}\to \mathrm{Cu}{\left(\mathrm{OH}\right)}_{4}^{-}+\;\bar{e}\;\mathrm{Ep}\;=750\;\mathrm{mV}\;\mathrm{vs}.\;\mathrm{Hg}|\mathrm{HgO}$$

(13) $$\mathrm{CuO}+H_{2}O+2{\mathrm{OH}}^{-}\to \mathrm{Cu}{\left(\mathrm{OH}\right)}_{4}^{-}+\;\bar{e}\;\mathrm{Ep}\;=750\;\mathrm{mV}\;\mathrm{vs}.\;\mathrm{Hg}|\mathrm{HgO}$$

As can be deduced, from the abovementioned consideration, copper may form various chemical products at various oxidation states when it is electrochemically oxidized. This is in opposition to the anodization of the majority of transition metals. For example, anodizing aluminum provides only Al^3+^ species, namely Al_2_O_3_ and in some cases AlO(OH). Therefore, numerous copper anodization products, as well as the much more complex mechanism of growth, provide new areas to explore.

## 3. Strategies of Copper Anodization

**Figure 2:**
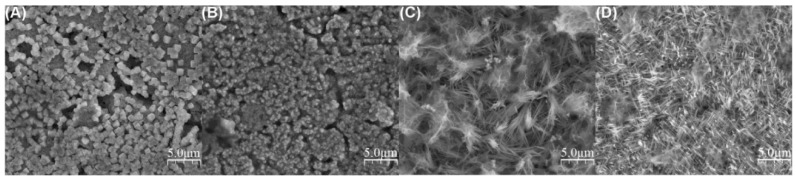
Top-view FE-SEM images of the surface morphology of the oxides formed via copper passivation in 1.0 M KOH at −400 (**A**); −300 (**B**); −200 (**C**); and −100 mV (**D**). Reproduced with permission from [41]. Elsevier, 2017.

**Figure 3:**
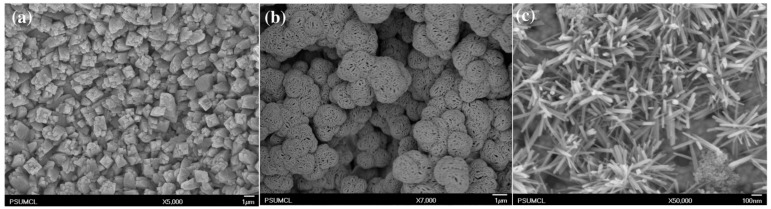
Top-view FE-SEM images of effects of Cu anodization in: (**a**) 0.15 M KOH + 0.1 M NH_4_Cl at 6 V for 300 s; (**b**) 0.2 M KOH + 0.1 M NH_4_F at 6 V for 300 s; and (**c**) aqueous solution of KOH (pH = 11) at 10 V. Reproduced with permission from [42]. Elsevier, 2011.

The passivation of copper, due to the abovementioned complexity, triggers numerous topics and needs for fundamental research. The easiest way to passivate electrochemically metal, that has not yet been explored in terms of anodization, is the application of a potentiostat with a three-electrode system, with which potentiostatic experiments in the passivity field are conducted according to Pourbaix diagram. Stępniowski et al. reported such a study on Cu passivation in 1 M aqueous solution of KOH [41]. The voltammetric study showed without any doubt two distinct oxidation peaks, at ca. −450 and −150 mV vs. Ag|AgCl, responsible for metallic copper oxidation to Cu^+^ and Cu^2+^, respectively. Moreover, the morphology of the obtained oxides strongly depends on the applied potential: for low potentials, micron-sized cubes were formed, mainly made of cuprous oxide, while at −200 and −100 mV vs. Ag|AgCl, nanowires were grown that were composed of a mixture of cuprous and cupric oxide (Figure 2). Nevertheless, a photoluminescence study revealed the presence of CuO on the surfaces of all of the samples, shedding some light on the growth mechanism. This suggests, that oxidation first occurs from Cu° to Cu^+^ and then, at the surface, Cu^+^ oxidizes to Cu^2+^. This is in line with the above-described considerations of anodic oxides’ growth on copper [40]. It is noteworthy that the formed nanowires grow gathered in bundles. For example, those obtained at −200 mV (Figure 2C) grew in bundles 72 ± 14 nm thick, while individual nanowires were 24 ± 5 nm thick. Those formed at −100 mV were gathered into bundles 90 ± 23 nm thick, while the individual nanowires were 19 ± 7 nm thick [41]. According to Allam and Grimes, the morphology of the grown oxide can be diverse, but it can be controlled by the operating conditions, including various additives to the electrolyte [42]. They applied a two-electrode system, using KOH with various salts as additives, in order to check the influence of the additives on the morphology of the formed oxide. It was found that nanoneedles formed in a pure aqueous solution of KOH (pH = 11), while an addition of halogen salts such as NH_4_F and NH_4_Cl allowed the formation of micrometric crystals rather than nanostructures (Figure 3).

Furthermore, anodizing in ethylene glycol containing KOH and NH_4_F also did not allow the achievement of nanostructures, like those anodized in an aqueous electrolyte. Leaf-like structures were obtained instead. Table 1 summarizes exemplary anodizing recipes in KOH-based electrolytes.

Generally, anodization in aqueous KOH electrolytes results in the formation of nanoneedles [42,45,46,47], nanowires [41], and nanorods [44]. Specifically, in copper anodization, nanoneedles are as long as nanowires but their diameter decreases close to their top, while nanowires have quite a uniform diameter along their length (nanorods are like nanowires, but with a much smaller aspect ratio). A wide range of the structures’ diameter has been observed; while Stepniowski et al. reported the smallest nanowire diameter equal 19 nm [41], Xiao et al. [46] reported the formation of nanoneedles with a diameter of 170 nm (up to 10 µm long) and the formation of nanoneedles 500–550 nm thick was reported by Wu et al. [47]. Thus, this shows that the anodization of copper provides the possibility to tune the morphology of the grown oxides in a wide range, making this technique competitive in comparison to others. Numerous other techniques have limitations in morphology control, while anodization allows to one obtain high-aspect ratio one-dimensional (1D) nanostructures within a wide range of diameter.

Additives, compounds added to the electrolytes, allow the modification of the morphology, as detailed in the abovementioned publication [42]. It is worth noting that recently a manufactured nanoporous material made of Cu_2_O, CuO, Cu(OH)_2_, and CuF_2_ was obtained with a pore diameter ranging from 6 to 15 nm [49]. Typically, the majority of the anodic oxides formed on copper are made of nanowires, nanoneedles, or nanorods. Nanoporous morphology is typical for oxides grown on other metals. Moreover, the pores formed via copper anodization have smaller diameters than the majority obtained by aluminum anodization. However, the ordering of the pores formed on copper is much poorer, compared to highly-ordered AAO. Nevertheless, the formation of porous oxide on copper confirms the vast variety of morphologies possible to obtain by anodization.

It is also worth noting that, analogous to aluminum anodization, and in the case of copper anodization, both potentiostatic and galvanostatic approaches are used. At a constant voltage (potentiostatic approach), the voltage resulting in the formation of anodic oxides in KOH-based solutions ranges from 4 to 30 V in two-electrode systems. When the galvanostatic approach is used, the current density ranges from 0.5 to 4.0 mA/cm^2^ (Table 1). Due to the alkaline hydrolysis, potassium oxalate was also successfully applied in copper anodization [43], producing a microporous mixture of CuO and CuO*_x_* due to the appropriate pH of the applied electrolyte (compare to the Pourbaix diagram, Figure 1). Thus, this shows there is still much to explore in the anodization of copper. THe Pourbaix diagram shows numerous opportunities for copper anodization at a more ambient pH. Carbonates and bicarbonates of alkali metals would be ideal for further fundamental research in this field.

**Figure 4:**
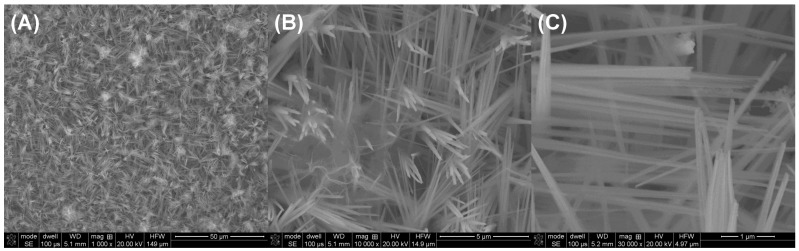
FE-SEM images of nanostructures grown on copper in 1.0 M NaOH at −200 mV vs. Ag|AgCl for 10 min at room temperature (RT). Images taken at different magnifications (**A**–**C**). Unpublished research by Stępniowski et al.

Wu et al. recently reported the behavior of copper in 0.1 M NaOH during cyclic voltammetry scans. According to their findings, copper in NaOH oxidizes in a few stages: at low potentials, Cu_2_O is first formed, then Cu_2_O at the surface is oxidized to CuO, and finally, Cu(OH)_2_ forms the outer layer of the oxide structures [50]. These findings are analogous to the recent results for Cu oxidation in 1 M KOH [41] and they are also in line with the Pourbaix diagram, where at lower potentials copper oxidized to Cu_2_O and then, at greater potentials, oxidized to CuO (Figure 1). Anodization in NaOH-based solutions also allows the formation of various nanostructures. The most desired nanostructures are nanoneedles and nanowires, due to their high surface area, like those formed in 1 M NaOH (Figure 4). Table 2 summarizes the recipes for copper anodization in NaOH-based solutions.

According to Table 2, it is apparent that various approaches were tested to grow anodic oxides on Cu in NaOH-based electrolytes. Here also the three-electrode approach using cyclic voltammetry [50,53] or constant potential [51,55,57] was applied. In a two-electrode approach, galvanostatic anodizing was applied with current densities in the range of 0.06 mA/cm^2^ [52] up to 5 mA/cm^2^ [54,58]. The concentration of NaOH in the applied electrolyte was used in the range from 0.1 [50,51] to 3.0 M [54], but also in this case, various additives were used to grow the nanostructured oxides, namely: NaCl with ethylene glycol [56], NaClO_2_ in order to enhance oxidation [57], and NH_4_Cl [58]. The application of sodium hydroxide-based electrolytes allowed the achievement of oxides with various morphologies, such as nanoparticles [51,53,55], nanowires [52,54], dendrites [53], nanoneedles [50], and nanosheets [56]. Also in this case, copper anodization offered a wide range of nanoneedle diameters and a high aspect ratio, overcoming the limitations of other techniques (Table 2).

In further applications, especially in physical ones such as photovoltaics, chemical cleanness is crucial. Unfortunately, in the majority of the research, detailed chemical composition analyses, mainly X-ray photoelectron spectroscopy (XPS), have revealed the simultaneous presence of Cu_2_O, CuO, or even Cu(OH)_2_ in the grown nanostructures [56,57,58]. For energy harvesting applications, such as PV (photovoltaics) or photocatalytic water decomposition, Cu_2_O is the most demanded among the copper species and its nanostructured form provides numerous surpluses thanks to its high surface area. To face the challenge of chemical composition homogeneity, Zhang et al. reported three methods of transforming Cu(OH)_2_ nanostructures into Cu_2_O [58]. One of them was hydrolysis with hydrogen peroxide (14):

(14) $$2\mathrm{Cu}{\left(\mathrm{OH}\right)}_{2}+2H_{2}O_{2}\to {\mathrm{Cu}}_{2}O+O_{2}+3H_{2}O$$

Another involved the annealing of the anodized samples in hydrogen at 280 °C (15):

(15) $$2\mathrm{Cu}{\left(\mathrm{OH}\right)}_{2}+2H_{2}\to {\mathrm{Cu}}_{2}O+3H_{2}O$$

And the third involved a reaction with glucose (used in electrochemical glucose sensing) that allowed the reduction of Cu(OH)_2_ to Cu_2_O (16):

(16) $$2\mathrm{Cu}{\left(\mathrm{OH}\right)}_{2}+{\mathrm{CH}}_{2}-{\left(\mathrm{CHOH}\right)}_{4}-\mathrm{CHO}\to {\mathrm{Cu}}_{2}O+{\mathrm{CH}}_{2}-{\left(\mathrm{CHOH}\right)}_{4}-\mathrm{COOH}+2H_{2}O$$

The obtained cuprous oxide had improved ability in photocatalytic oxygen generation from water, although the best results (the most efficient oxygen production) were achieved with the samples treated with hydrogen peroxide. This means that the reduction of copper from Cu(II) to Cu(I) was the most efficient. Therefore, the nanostructures obtained by Cu anodization can be chemically reduced, forming high-surface area material made of Cu_2_O. Such numerous options of cuprous oxide as well as cupric oxide nanostructuring allow researchers to apply these nanostructures in various devices.

## 4. Properties and Applications of Anodic Nanostructures Grown on Copper

The high surface area and chemical composition that could be tailored via anodization encouraged scientists to apply these materials in areas such as tunable contact angle surfaces, sensing, and renewable energy harvesting.

As mentioned above, the copper species formed via anodization are at various oxidation states. Also, chemical post-treatments can be applied to oxidize or reduce the formed nanostructures. Thus, they can form redox couples with various chemical compounds, providing reactions that can be electrochemically sensed. One such reaction is glucose oxidation to gluconic or glucuronic acid (see Equation (16)). However, the C–C bonds in glucose may undergo dissociation during the electrochemical oxidation and form shorter products like formats, or even carbonates. Simultaneously, active surface Cu(III) species are reduced to Cu(II) [43]. Satheesh Babu and Ramachandran anodized Cu in potassium oxalate in order to obtain an electrochemical glucose sensor [43]. This allowed the formation of CuO with a developed surface area, which is desired in sensing applications. Applied potassium oxalate solution as the electrolyte allowed the incorporation of oxalates into the CuO, in the form of Cu(II) oxalate. This in situ doping approach has already been intensively researched for other anodic oxides, like alumina [37]. However, it was shown in this work [43] that the in situ doping of CuO is also possible and beneficial for sensing applications. The electrochemical sensor made of Cu coated with CuO and copper oxalate was found to have a sensitivity as high as 1.89 mA·mM^−1^·cm^−2^ with a detection limit equal to 50 nM. Additionally, the sensor was found to work in the presence of other interfering compounds like ascorbic acid and uric acid, as well as in samples of human blood serum, confirming its outstanding selectivity towards glucose.

**Figure 5:**
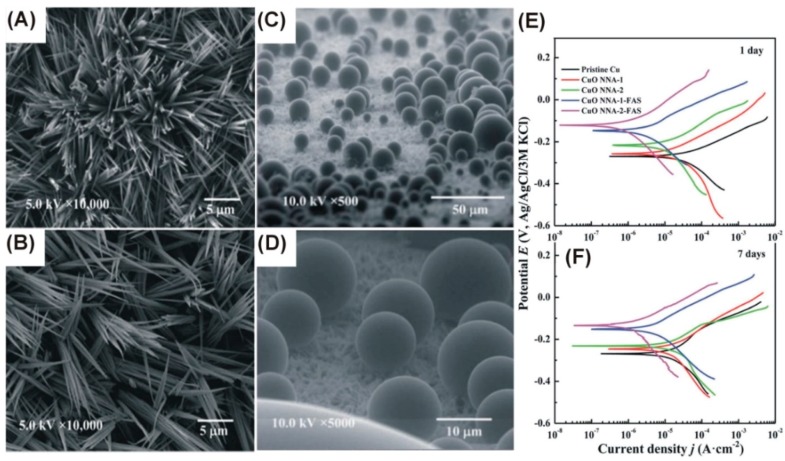
Top-view FE-SEM images of CuO nanoneedles formed in 2 M KOH at 15 °C, 2 mA·cm^−2^ for 25 (**A**) and 40 min (**B**); behavior of 3.5% NaCl solution on their surface after the chemical bonding of FAS-17 (**C**,**D**) and their corrosion performance after 1 (**E**) and seven days of immersion in 3.5% NaCl (**F**). CuO NAA-1 and CuO NAA-2 indicate anodization for 25 and 40 min, while FAS denotes the subsequent modification with FAS-17. Reproduced with permission from [46]. Royal Society of Chemistry, 2015.

Due to the developed surface area and diverse morphologies of the formed anodic films, the modification of the wetting of the surface can be provided by copper anodization. There are two main approaches describing the behavior of liquids on surfaces with nanostructures on the top (and mixed approaches as well): Wenzel’s approach, in which the nanostructures are deeply penetrated by the liquid, and Cassie-Baxter’s method, in which the liquid does not penetrate the nanostructures and air remains trapped inside them. According to Jiang et al., the nanostructures grown via copper anodization are in the Cassie-Baxter state [52]. They anodized copper in 1 M NaOH (Table 2) and obtained Cu(OH)_2_ nanowires that were hydrophilic (wetting contact angle was 4.5° and 0° for water and CH_2_I_2_, respectively). Due to the provided modification of the surface with the chemical bonding of FAS-17 (2*H*,2*H*-Perfluorodecyltriethoxysilane), the contact angle increased significantly to 154° and 133° for water and CH_2_I_2_, respectively [52]. FAS-17-modified CuO nanostructures were also used to research anti-corrosion applications of the as-formed hydrophobic coating. Xiao et al. reported the formation of CuO nanoneedles in KOH that were 7–10 µm long and ca. 170 nm in diameter (Table 1) (Figure 5A,B) [46]. Post-treatment with FAS-17 allowed the increase of the wetting water contact angle up to 169°, providing superhydrophobicity (Figure 5C,D). Furthermore, the limited contact between the surface and surrounding liquid environment (Cassie-Baxter state) hinders galvanic coupling with the material underneath, consequently improving the corrosion performance. After Cu anodization for 40 min in 2 M KOH at 2 mA·cm^−2^ at 15 °C, the corrosion potential increased from −254 to −212 mV, while the corrosion current density recorded in 3.5% NaCl decreased from 19.58 µA·cm^−2^ to 9.11 µA·cm^−2^ [46]. Further surface chemical modification by FAS-17 bonding increased the hydrophobicity and consequently increased the corrosion potential to −124 mV and decreased corrosion current to 0.66 µA·cm^−2^. After one week of immersion in 3.5% NaCl, the corrosion performance of the anodized and chemically modified copper remained satisfactory: the corrosion current density was then 0.99 µA·cm^−2^ and the corrosion potential was −134 mV (Figure 5E,F). Thus, morphologically and chemically generated hydrophobicity could hinder the charge transfer at the electrolyte-sample interface, as confirmed by electrochemical impedance spectroscopy (EIS)-derived charge transfer resistance, which was 2.47 kΩ·cm^2^ for anodized and modified samples vs. 0.25 kΩ·cm^2^ for pristine Cu and 0.44 kΩ·cm^2^ for the only anodized surface.

**Figure 6:**
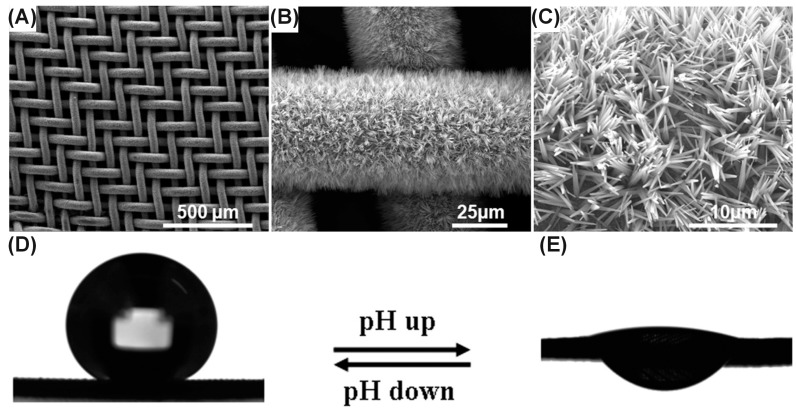
SEM images of Cu mesh subjected to anodization in 2 M KOH at 1.5 mA·cm^−2^ (**A**–**C**) and water permeation performance after anodization, Au sputtering, and functionalization with thiols (**E**,**F**); water droplet size was 4 µL (**D**,**E**) and pH was 2 (**D**) and 12 (**E**). Reproduced with permission from [45]. American Chemical Society, 2012.

Another application, using the superhydrophobic effect admitted to the morphology of anodic oxides grown on copper, employs a pH-responsive water permeation mesh [45]. Cu(OH)_2_ nanoneedles formed in KOH (see Table 1 and Figure 6A–C) were coated with gold and then thiols, HS(CH_2_)_9_CH_3_ and HS(CH_2_)_10_COOH, were chemically bonded to the surface. At a low pH, such a nanostructured mesh had a contact angle equal 153°, which did not allow water to pass through the mesh (Figure 6D). However, increasing pH to 12 significantly decreased the contact angle (ca. 8°) and consequently the mesh was permeable to water [43]. What is even more interesting, the pH-response time was about 3 s. Moreover, this system was reversible and switching the pH allowed one to switch contact angle and, consequently, the permeability. It is also important to note that the starting material had a complex geometry in the micro scale (mesh) and it was successfully anodized. Therefore, this paper shows that samples with sophisticated morphology can be anodized, providing cuprous and cupric oxide nanostructures on the surface. Typically, when Al or Ti are anodized, the starting material samples are plates or rods. Very often, due to the complex geometry of the metallic substrate, anodization is impossible because the anodic dissolution of metals occurs at the exposed edges of the sample. Reporting the anodization of Cu mesh enables numerous opportunities in other applications, where already the starting material must have complex geometry, but formation of surface nanostructures would be beneficial and in-demand.

One of the applications in which cupric oxide nanostructures formed via anodization may bring significant input is renewable energy harvesting [59]. Cu_2_O (a p-type semiconductor) coupled with ZnO (an n-type semiconductor) can be used as an efficient hetero-junction photovoltaic cell. However, so far only around a 6% power conversion efficiency (PCE) of Cu_2_O-based photovoltaic cells has been reported, versus 23% of the theoretical PCE value [59]. Numerous nanostructuring approaches, including the above-discussed anodization, may bring progress in the PCE. Due to the electronic structure of Cu_2_O and CuO, nanostructures obtained by copper anodization will find applications in renewable fuel generation, namely in photoelectrochemical (PEC) water splitting. The semiconducting photocathode has a suitable band gap for water splitting reactions, which is within the band gap of Cu_2_O and CuO. 

It is important to note that the band gap of CuO is smaller than that of Cu_2_O; moreover, both the valence and conduction bands of CuO are lower than those of Cu_2_O. Briefly, after the excitation of the photocathode made of Cu_2_O-CuO film, when the excited electron will exceed the band gap of Cu_2_O, it can reduce its energy down to the conduction band of CuO and by further reduction cause the decomposition of water, generating gaseous hydrogen and oxygen. Nevertheless, one should be aware that corrosive reactions, like Cu_2_O or CuO reduction, may occur as well, affecting the performance of the photocatalyst over time. Zhang et al. reported an extensive study on copper anodization for photoelectrochemical water splitting [56]. They investigated a few strategies of copper anodization, employing various current densities and post-treatments such as post-annealing, causing calcinations and improving the crystallinity of the formed nanoneedles (Table 2). The reported research confirmed that the mixed oxide nanoneedles, consisting of a Cu_2_O-CuO system, have the best performance towards PEC water splitting. The greatest photocurrents were recorded for Cu/Cu_2_O/CuO and Cu/Cu_2_O/Cu(OH)_2_, namely −1.54 and −1.28 mA/cm^2^, respectively, at no external voltage (0 vs. NHE — Normal Hydrogen Electrode). Nevertheless, due to the mentioned corrosion of cupric and cuprous oxide, their stability dropped over time. Cu/Cu_2_O/CuO and Cu/Cu_2_O/Cu(OH)_2_ preserved after 20 min represented 74.4% and 85.8% of their performance, respectively. Pure Cu_2_O film, after 20 min of performance, had only 30.1% of its primal conversion efficiency (simultaneously harvesting a much lower current density: −0.65 mA/cm^2^). 

Photocatalytic oxygen generation is another method of renewable energy harvesting, enabling the storage of energy. In order to generate oxygen from water, photo-generated holes have to recombine on the surface of the catalyst, according to Equation (17) [58]:

(17) $$2H_{2}O+4h^{+}\to O_{2}+4H^{+}$$

This reaction lays in the band gap of Cu_2_O, thus nanostructures formed by Cu anodization are also suitable for this application. However, in contrast to PEC water splitting, in this case the presence of CuO would affect the yield of the reaction. Thus, the authors [58] worked out three various approaches to reduce Cu(II) to Cu(I), mentioned in the previous section (Equations (14)–(16)). According to the authors, the oxygen generation reaction occured efficiently due to the contact of metallic Cu with Cu_2_O, as it allowed the rapid acceptance of electrons generated by metal, with a simultaneous rapid conversion of holes on the catalysts’ surface. Thus, the formation of charge imbalance was hindered due to this junction. Among three post-treatment methods, hydrolysis with hydrogen peroxide obtained the greatest oxygen production yield, providing 233.27 µmol from 1 mg of catalyst in 8 h of its performance, while methods obtained results below 185 µmol of O_2_ [58]. 

On the other hand, the post-treatment of the grown anodic oxides with KMnO_4_ was reported by Arurault et al. [57]. This enabled the oxidization of the grown anodic film to CuO and Cu_3_O_2_. The highly-developed surface area combined with the chemical composition increased solar light absorption. Thus, it was demonstrated that anodic oxides grown on copper are suitable for solar cell applications.

A brief review of anodic oxides grown on copper with their applications is presented in Table 3. It leads to the conclusion that the major applications of anodic oxides grown on copper result from their chemical composition and nanostructured morphology. However, most applications are reported for anodic oxides in the form of nanoneedles. This form of nanostructure provides high-surface area for photocatalytic reactions high wetting contact angle (after functionalization), and high sensitivity.

## 5. Anodization versus Other Nanostructured Copper Oxide Fabrication Methods

According to the abovementioned applications, 1D cuprous and cupric oxide nanostructures may bring valuable contributions to many fields. Thus, a variety of the Cu_2_O and CuO synthesis techniques are being developed by researchers. The literature study shows that the self-organized anodization of copper may be attractive for few reasons (Table 4):

- It employs inexpensive equipment;
- It is time-efficient;
- It is an easy technique to scale-up, as it employs self-organization (no template required);
- It does not require numerous steps, only electropolishing and one step of the electrochemical oxidation;
- It allows for the control of geometry and provides 1D nanostructures with a high aspect ratio.

On the other hand, there are numerous competitive chemical techniques, like precipitation, sol-gel, or hydrothermal synthesis, but 1D nanostructures obtained with those methods have much smaller aspect ratios. Moreover, the chemical syntheses are multistep processes. Additionally, some of the steps take tens of hours to complete. Template-assisted techniques that also employ atomic layer deposition (ALD) may provide high aspect ratio 1D nanostructures made of cuprous and cupric oxides; however, these methods are also multistep (the formation of template also must be taken into account) and employ expensive tools (ALD).

Therefore, the self-organized anodization of copper, resulting in the formation of 1D nanostructures, may nowadays attract much more attention due to the offered solutions and potential applications of the nanostructures.

## 6. Conclusions and Challenges

The self-organized anodization of copper allows one to obtain anodic oxides with various morphologies, including nanoneedles, nanowires, or even nanoporous structures. Furthermore, the chemical composition of the formed nanostructures can be varied using appropriate experimental conditions in anodizing and various post-treatment procedures. The literature reveals that nanostructured oxides grown on copper can contribute to glucose sensing in the presence of interfering compounds and in human blood serum. Furthermore, the nanoneedles grown by copper anodization are capable of forming superhydrophobic surfaces, including smart, pH-responsive permeation systems. Copper anodization also contributes to renewable energy harvesting research: it enables the photocatalytic decomposition of water to hydrogen and oxygen. When the photocatalyst is made of a Cu_2_O-CuO mixture, it can generate oxygen from water.

The current state-of-the art review also revealed numerous challenges in copper anodization:

1. There are challenges in the formation of anodic copper oxides: generally, only KOH- and NaOH-based electrolytes were used for anodizing. The Pourbaix diagram revealed many more opportunities. Thus, electrolytes in a pH range from ca. 7 to 11 could provide different morphologies and more uniform chemical compositions. Potassium oxalate, used as an anodizing electrolyte, shows that there is still much to explore in this field.
2. A full systemic study of the influence of the operating conditions (type of the electrolyte, voltage, time, temperature) on the morphology of the grown nanostructures has not yet been reported, though this would be beneficial for all researchers working in the field of applications of copper and cuprous oxide nanostructures. In this field, the formation of nanostructures via anodization with a diameter smaller than ca. 7 nm, in order to observe a significant QC effect, would be also challenging.
3. There is also a demand to quantify the chemical composition of the grown nanostructures versus the operating conditions of anodization. It would be beneficial from both a fundamental point of view (understanding the mechanism of growth with hard, experimental data) and for applications in which chemical purity is crucial, such as photovoltaics.
4. In contrast to all anodic oxides, nanostructures grown by copper electrochemical oxidation are crystalline. From a fundamental point of view, investigating the crystal orientation would be important, so as to determine whether the anodization of a planar sample, in a constant electric field, induces the growth of oriented, crystalline nanowires and nanoneedles.

In summary, anodization, a relatively novel approach in copper oxides nanoengineering, allows one to obtain a variety of morphologies, thus contributing to state-of-the-art emerging applications.

## Figures and Tables

**Table 1:** Gathered experimental conditions for nanostructures formed via copper anodization in KOH-based solutions.

Chemical Composition of the Electrolyte	Experimental Conditions	Morphology and Chemical Composition of the Oxide	Remarks	Reference
0.25 M 0.5 M 1.0 M K_2_C_2_O_4_ aq.	Cyclic voltammetry: 50 mV/s Range: −1.0 to 0.8 V RT ^1^	CuO/CuO*_x_* Microporous structure	Anodized surface was used as an electrochemical sensor of glucose; glucose was determined at concentrations as low as 4 mM in human blood serum using anodized copper	Satheesh Babu 2010 [43]
Aqueous KOH	4–6 V RT	Nanoneedles	No nanostructures were formed in aqueous KOH at pH < 10	Allam 2011 [42]
Aqueous KOH	10 V, pH = 11, 11.5, 12	Nanorods	At a pH below 10, Cu was dissolved; light blue precipitate was formed on the samples; surface nanostructuring enhanced the photoelectrochemical response	Shooshtari 2016 [44]
2 M KOH	1.5 mA/cm^2^, varied duration	Cu(OH)_2_ nanoneedles	Cu mesh was anodized in order to change the contact angle (CA); CA was pH-responsive: the lower the pH, the greater the CA, up to 153°	Cheng 2012 [45]
0.5–4.0 M KOH	0.5–4.0 mA/cm^2^, 5–25 °C, 25 min	CuO nanoneedles 170 ± 40 nm in diameter 7–10 µm long	Fluoroalkyl-silane (FAS-17) was chemically bonded to CuO nanoneedles, increasing the contact angle up to 169°	Xiao 2015 [46]
2, 2.5, 3, 3.5 M KOH	1.5 mA/cm^2^, 2, 15, 28 °C	Cu(OH)_2_ and CuO nanoneedles 500–550 nm in diameter	Cu(OH)_2_ were turned into CuO nanoneedles using heat treatment (150 °C at 3 h + 200 °C at 3 h); in 3 M KOH at 28 °C nanotubes were formed (80–500 nm diameter, 10 µm length)	Wu 2005 [47]
1 M KOH	−0.4, −0.3, −0.2, −0.1 V vs. Ag|AgCl, RT, 1 h	Morphology depends on the potential; cubes and nanowires	Formed nanowires were mixtures of Cu_2_O and CuO; nanowires were obtained for −0.2 (24 nm in diameter) and −0.1 V (19 nm in diameter), while for −0.4 and −0.3 V micro-cubes were formed	Stepniowski 2017 [41]
0.15 M KOH + 0.1 M NH_4_Cl	6 V, 300 s, RT	Cubes and dendrites	-	Allam 2011 [42]
0.15 M KOH + 0.1 M NH_4_F	6 V, 300 s, RT	Cu_2_O Micro-balls made of whiskers forming nanopores	^3^ XPS and ^4^ GAXRD proved that the structures were made of Cu_2_O	Allam 2011 [42]
0.15 M KOH + 0.1 M NH_4_F + 3% H_2_O in EG ^2^	30 V, 300 s, RT	160-nm thick leaf-like architectures	When KOH concentration was increased to 0.2 M, the leaf-like structures were ca. 500 nm thick; at voltages below 30 V, no structured film was formed	Allam 2011 [42]
0.75 wt % KOH + 3 wt % H_2_O + 0.20–0.35 wt % NaF in EG	10–30 V, 10 min	Cu_2_O film	Cu_2_O film was formed by anodization; further annealing (250–450 °C, 60 min) allowed the growth of CuO nanowires, improving the photoelectrochemical performance	Wang 2013 [48]
0.1–0.5 M KOH + 0–0.1 wt % NH_4_F + 1 vol % H_2_O in EG	5–20 V, 5 °C	Nanoporous film (6–15 nm pore diameter)	Nanoporous oxide was formed, composed of a mixture of the following species: Cu_2_O, CuO, Cu(OH)_2_, and CuF_2_	Oyarzún Jerez 2017 [49]

^1^ RT—room temperature; ^2^ EG—ethylene glycol; ^3^ XPS—X-ray photoelectron spectroscopy, ^4^ GAXRD—glancing angle X-ray diffraction.

**Table 2:** Gathered experimental conditions for nanostructures formed via copper anodization in NaOH-based solutions.

Chemical Composition of the Electrolyte	Experimental Conditions	Morphology and Chemical Composition of the Oxide	Remarks	Reference
0.1 M NaOH	−400 mV, 1 h	Nanoparticles	Mechanism of oxide growth was studied	Caballero-Briones 2010 [51]
0.1 M NaOH	10 mV/s voltammetric scan from −1.2 to 0.8 V RT	Cu needle was anodized and coated by oxide-hydroxide film	Mechanism of Cu electrochemical oxidation was investigated	Wu 2013 [50]
1 M NaOH	0.06 mA/cm^2^, 5 min, 25 °C	Cu(OH)_2_ nanowires	Nanowires surface was modified by the chemical bonding of 1*H*,1*H*,2*H*,2*H*-Perfluorodecyltriethoxysilane (FAS-17) in order to increase the wetting contact angle to 154°; as-obtained nanowires were made of Cu(OH)_2_, but further annealing enabled the transofrmation of the hydroxide into CuO	Jiang 2015 [52]
1 M NaOH	Cyclic voltammetry from −1.6 V to 0.4 V RT	CuO dendrite crystals grown on Cu_2_O nanoparticles	Cyclic voltammetric study of Cu in alkaline solution	Wan 2013 [53]
3 M NaOH	1.5, 3.0, 5.0 mA/cm^2^, 30 min	Cu_2_O, Cu_2_O/Cu(OH)_2_, Cu(OH)_2_ nanowires	Cu was electrodeposited on ITO (Indium Tin Oxide) and subsequently anodized; obtained nanostructures enhanced photocatalytic water splitting; the best results were achieved for nanowires made of both Cu_2_O and CuO	Zhang 2012 [54]
0.15 M NaOH	pH = 12.8–13.0 −430 mV	Cu_2_O nanoparticles	Cu_2_O behaved like a p-type semiconductor	Caballero-Briones 2009 [55]
1 M NaOH + 2.5 M NaCl + 0.5 g/L EG	0.5–2.5 A/dm^2^, 55–70 °C, 30 min	30-nm thick nanosheets made of CuO and Cu_2_O	Formed nanostructures are mixtures of CuO and Cu_2_O	Shu 2017 [56]
10 wt % NaOH + 5 wt % NaClO_2_	0.75 V, 60 °C, 15 min	CuO films with traces of Cu_3_O_2_, decomposing to CuO and Cu_2_O	After anodizing, samples were immersed in KMnO_4_; consequently, solar light absorption reached up to 96%	Arurault 2007 [57]
0.2 M NH_4_Cl; pH was adjusted to 8 with NaOH	5 mA/cm^2^, RT, 20 min	Cu(OH)_2_ film	In order to transform Cu(OH)_2_ into Cu_2_O, three post-treatments were applied: hydrolysis in H_2_O_2_, reduction in H_2_ at 280 °C, redox with glucose; the H_2_O_2_ post-treated sample had the best efficiency in oxygen generation	Zhang 2015 [58]

**Table 3:** Gathered information about applications of nanostructures formed via copper anodization.

Application	Role of the Anodically Grown Nanostructures	Remarks	Reference
Glucose sensor	High surface area Cu(III) active sites were reducted to Cu(II) by glucose	Glucose was determined at concentrations as low as 4 mM in human blood serum	[43]
pH-responsive water permeation mesh	High surface area	Wetting contact angle was pH-switchable and for a lower pH it reached up to 153°	[45]
High contact angle surface for corrosion protection	Highly-developed nanostructured surface area	Fluoroalkyl-silane (FAS-17) was chemically bonded to CuO nanoneedles, increasing the contact angle up to 169°; the corrosion performance was significantly improved	[46]
High contact angle surface	Highly-developed nanostructured surface area	Nanowires‘ surface was modified by the chemical bonding of 1*H*,1*H*,2*H*,2*H*-Perfluorodecyltriethoxysilane (FAS-17) in order to increase the wetting contact angle to 154°	[52]
Photoelectrochemical chemical water splitting	Highly-developed surface area and chemical composition (CuO-Cu_2_O)	Nanostructures enhanced photocatalytic water splitting; the best results were achieved for nanowires made of both Cu_2_O and CuO	[54]
Photochemical oxygen generation	High surface area and chemical composition (Cu_2_O)	Post-treatment of obtained nanostructures was conducted	[58]
Solar light absorption	Chemical composition (CuO with Cu_3_O_2_)	Post-treatment in KMnO_4_ was conducted	[57]

**Table 4:** Drawbacks of currently applied copper oxides nanostructuring methods vs. solutions offered by anodizing.

Fabrication Method	Drawbacks of the Method	Solution offered by Anodizing	Reference
Hydrothermal synthesis	Small aspect ratio of one-dimensioanl (1D) nanostructures; requires a few steps of synthesis	High aspect ratio of 1D nanostructures can be easily achieved by lengthening the time of anodization; facile, easy-to-scale-up two-step synthesis (electropolishing + anodizing)	[35,36]
Atomic layer deposition	Requires templates and expensive equipment to grow 1D nanostructures	Method based on easy to scale-up self-organization; inexpensive, electrochemical method	[35,36]
Solution-based chemical precipitation	Small aspect ratio of 1D nanostructures	Relatively high aspect ratio (see Table 1 and Table 2)	[35,36]
Template techniques (i.e., Anodic Aluminum Oxide, AAO and subsequent deposition)	Numerous steps of synthesis (formation of template, deposition, removal of the template)	An easy-to-conduct, two-step synthesis (electropolishing + anodizing)	[59]
Sol-gel techniques	Multistep process, time-consuming method	Anodization can be minutes long, in order to achieve a surface covered by oxide nanoneedles	[60]
